# The last frontiers of wilderness: Tracking loss of intact forest landscapes from 2000 to 2013

**DOI:** 10.1126/sciadv.1600821

**Published:** 2017-01-13

**Authors:** Peter Potapov, Matthew C. Hansen, Lars Laestadius, Svetlana Turubanova, Alexey Yaroshenko, Christoph Thies, Wynet Smith, Ilona Zhuravleva, Anna Komarova, Susan Minnemeyer, Elena Esipova

**Affiliations:** 1 University of Maryland, College Park, MD 20740, USA.; 2 Laestadius Consulting LLC, Silver Spring, MD 20901, USA.; 3 Greenpeace Russia, Moscow, Russia.; 4 Greenpeace Germany, Hamburg, Germany.; 5 Global Forest Watch Canada, Ottawa, Ontario, Canada.; 6 World Resources Institute, Washington, DC 20002, USA.; 7 NGO Transparent World, Moscow, Russia.

**Keywords:** Wildlands, forest, biodiversity, carbon storage, intactness, ecosystem services, remote sensing, intact forest landscapes

## Abstract

An intact forest landscape (IFL) is a seamless mosaic of forest and naturally treeless ecosystems with no remotely detected signs of human activity and a minimum area of 500 km^2^. IFLs are critical for stabilizing terrestrial carbon storage, harboring biodiversity, regulating hydrological regimes, and providing other ecosystem functions. Although the remaining IFLs comprise only 20% of tropical forest area, they account for 40% of the total aboveground tropical forest carbon. We show that global IFL extent has been reduced by 7.2% since the year 2000. An increasing rate of global IFL area reduction was found, largely driven by the tripling of IFL tropical forest loss in 2011–2013 compared to that in 2001–2003. Industrial logging, agricultural expansion, fire, and mining/resource extraction were the primary causes of IFL area reduction. Protected areas (International Union for Conservation of Nature categories I to III) were found to have a positive effect in slowing the reduction of IFL area from timber harvesting but were less effective in limiting agricultural expansion. The certification of logging concessions under responsible management had a negligible impact on slowing IFL fragmentation in the Congo Basin. Fragmentation of IFLs by logging and establishment of roads and other infrastructure initiates a cascade of changes that lead to landscape transformation and loss of conservation values. Given that only 12% of the global IFL area is protected, our results illustrate the need for planning and investment in carbon sequestration and biodiversity conservation efforts that target the most valuable remaining forests, as identified using the IFL approach.

## Teaser

Intact forest landscapes have the greatest conservation value but are shrinking due to industrial logging and fragmentation.

## INTRODUCTION

Human modification of terrestrial ecosystems has a range of impacts, from a complete transformation at a local scale to distant effects such as the impact of global climate change on ecosystem functions and dynamics (*1*, *2*). No ecosystems may be considered truly intact because some degree of human impact is present everywhere (*3*). Alteration and fragmentation of forest landscapes compromise their ecosystem functions, including loss of biological diversity and reduction of carbon storage (*4*, *5*).

Forest wildlands, those forests least affected by human activity, have the highest conservation value in terms of the range of ecosystem services they provide (*6*–*10*). These areas are often irreplaceable in harboring biological diversity, stabilizing terrestrial carbon storage, regulating hydrological regimes, and providing other ecosystem functions (*11*). Their ability to perform ecosystem functions and their resilience to natural disturbance and climate change are functions of their size. Many “umbrella” mammal and bird species, whose conservation also may enhance the protection of co-occurring species, require large natural habitats to survive (*12*). Large forest wildlands are the greatest terrestrial carbon stores, a function at risk from forest conversion (deforestation) and degradation (*10*). Small forest areas, even if pristine, have less potential for preserving wide-range species populations and have lower resilience to natural disturbance and effects of climate change (*4*). Hence, the size of the wildland matters: the larger the size, the higher the conservation value of the territory.

Preservation of forest wildlands requires a robust mapping and monitoring system that can be implemented at national to global scales. A number of global ecosystem wilderness and intactness maps have been created over the past 30 years (*3*, *13*, *14*). Most have relied on outdated, coarse spatial resolution and static input data, which may impede the accurate delineation of wilderness loss over time (*15*).

Delineating forest wildlands includes two components: assessing direct forest structural alteration (including forest conversion, timber extraction, and indirect effects, such as human-ignited fires) and the resulting fragmentation of the remaining forest landscapes due to such changes. Satellite data provide the most feasible solution for recurrent global mapping and monitoring of human-caused forest alteration and fragmentation (*16*).

We define an intact forest landscape (IFL) as a seamless mosaic of forests and associated natural treeless ecosystems that exhibit no remotely detected signs of human activity or habitat fragmentation and are large enough to maintain all native biological diversity, including viable populations of wide-ranging species (*15*). The global IFL mapping is based on a set of clear and straightforward criteria, designed to enable satellite-based mapping (see Materials and Methods). The term “intact forest landscape” is not congruent with the term “primary forest” as defined by the Food and Agriculture Organization of the United Nations (FAO) (*17*), and the two must not be confused. Primary forests are part of IFLs, which also include nonforest intact ecosystems where climatic, soil, or hydrological conditions prevent tree growth, temporally treeless areas after the natural disturbance (for example, wildfires), and water bodies. IFLs may also include areas affected by low-intensity and historic human influence, such as hunting, scattered small-scale shifting cultivation, and preindustrial selective logging. IFLs include large fragments of primary forests with a minimum extent of 500 km^2^, while smaller fragments of primary forests may be found outside IFLs. Here, we use the archive of Landsat satellite imagery to map the global extent of IFLs in the years 2000 and 2013, to locate changes due to alteration and fragmentation, and to identify causes of change.

## RESULTS

We assessed the distribution and dynamics of IFLs within the extent of present-day forest ecosystems. We defined “forest” as lands with a tree canopy cover greater than 20% in the year 2000, using a global tree canopy cover data set (*18*) as a reference. The present-day extent of forest landscapes (mosaics of forests, naturally treeless ecosystems, and deforested areas) is referred to as the “forest zone.” The forest zone extends over 58 million km^2^, or 44% of Earth’s ice-free land area. The extent of IFLs in the year 2000 totaled 12.8 million km^2^, or 22% of the forest zone area.

**Fig. 1:**
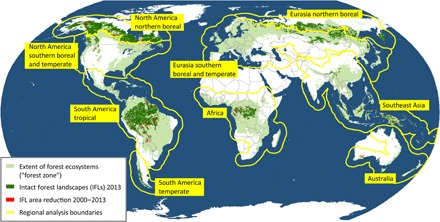
The world’s IFLs. IFL extent for the year 2013, IFL area reduction from 2000 to 2013, and boundaries of geographic regions used for the analysis.

The IFLs form distinctive regional groupings (Fig. 1 and Table 1), each with a unique history of alteration and fragmentation. In the humid tropics, IFLs are found in the Amazon and Congo River basins, the islands of Borneo and New Guinea, and the Southeast Asian highlands. Tropical regions comprise 48% of the total global IFL area. In dry tropical and subtropical regions, IFLs are scarce or absent due to extensive conversions to agriculture, some of which happened many centuries ago. Within the temperate and southern boreal forests of North America and Eurasia, IFLs remain only in small areas spared from commercial logging and agriculture. IFLs are abundant in northern boreal forests, interrupted mainly by mining, extraction of fossil fuels, and human-ignited wildfires associated with roads. Northern boreal IFLs comprise 36% of the total global IFL area.

**Table 1:** IFL extent and area reduction per geographic region.

**Geographic** **region**	**Forest** **zone area** **(km^2^ × 10^6^)**	**IFL 2000 area** **(km^2^ × 10^6^)**	**IFL proportion** **of the forest** **zone in** **2000 (%)**	*Forest **proportion** **within IFL** **2000 (%)**	**IFL proportion** **of global IFL** **area in** **2000 (%)**	**IFL** **2013 area** **(km^2^ × 10^6^)**	**IFL area** **reduction** **2000–2013 (%)**	**IFL area** **reduction** **2000–2013,** **not attributed** **to fire (%)**
Africa	9.08	1.00	11.0	99.8	7.8	0.90	10.1	10.1
Australia	1.01	0.13	12.4	55.6	1.0	0.10	21.9	15.3
South America, temperate	0.41	0.16	38.2	43.4	1.2	0.15	1.3	0.9
South America, tropical	14.70	4.43	30.1	98.9	34.6	4.11	7.3	7.1
North America, temperate and southern boreal	5.85	0.54	9.2	66.5	4.2	0.46	15.5	11.2
North America, northern boreal	3.89	3.04	78.2	63.8	23.7	2.94	3.3	0.3
Northern Eurasia, temperate and southern boreal	11.96	1.23	10.3	69.8	9.6	1.12	9.1	7.4
Northern Eurasia, northern boreal	3.33	1.57	47.0	75.7	12.2	1.50	4.4	1.8
Southeast Asia	7.38	0.72	9.8	93.7	5.6	0.62	13.9	13.9
World total	57.60	12.81	22.2	82.3	100.0	11.89	7.2	5.7

*Forest is defined here as land with tree canopy cover above 25%, as depicted by the global tree cover product (*18*).

IFLs were found within 65 countries in the year 2000 (Table 2). Three countries (Russia, Brazil, and Canada) account for nearly two-thirds of the global IFL area. These countries are followed by the Democratic Republic of the Congo, Peru, the United States (primarily Alaska), Indonesia, Colombia, and Venezuela, each contributing more than 2% to the global IFL area. French Guiana has the highest proportion of intactness of all countries, with IFLs making up 79% of the forest zone. This country is followed by Suriname, Guyana, Peru, Canada, Gabon, and the Republic of the Congo, each retaining more than 40% of their respective forest zone as IFLs in the year 2000.

**Table 2:** IFL extent and area reduction per country.

**Country name**	**Country code** **(for Fig. 3)**	**IFL 2000** **area (km^2^ × 10^3^)**	**IFL proportion** **of the forest** **zone in** **2000 (%)**	**IFL proportion** **of global IFL** **area in** **2000 (%)**	**IFL area** **reduction** **2000–2013 (%)**	**IFL area** **reduction** **2000–2013,** **not attributed** **to fire (%)**
Angola	AGO	2.9	0.3	0.02	13.7	13.7
Argentina	ARG	39.9	6.5	0.3	2.0	1.8
Australia	AUS	82.2	9.8	0.6	32.7	22.8
Belize	BLZ	4.3	19.7	0.03	4.8	4.8
Bhutan	BTN	6.4	19.3	0.05	15.5	15.5
Bolivia	BOL	233.3	28.9	1.8	19.6	18.3
Brazil	BRA	2476.1	31.7	19.3	6.3	6.2
Brunei	BRN	2.0	35.1	0.02	17.0	17.0
Cambodia	KHM	1.1	0.9	0.01	38.2	38.2
Cameroon	CMR	52.8	13.4	0.4	25.2	25.2
Canada	CAN	3040.3	51.0	23.7	4.7	2.3
Central African Republic	CAF	8.7	1.5	0.1	34.4	34.4
Chile	CHL	131.4	36.9	1.0	1.3	0.9
China	CHN	45.0	1.6	0.4	11.5	11.2
Colombia	COL	349.2	31.0	2.7	1.3	1.3
Costa Rica	CRI	3.2	6.2	0.02	3.0	3.0
Côte d’Ivoire	CIV	4.6	1.7	0.04	17.5	17.5
Cuba	CUB	0.5	0.5	0.004	0	0
Democratic Republic of the Congo	COD	643.9	27.7	5.0	4.2	4.2
Dominican Republic	DOM	0.8	1.7	0.01	29.0	1.6
Ecuador	ECU	53.3	22.3	0.4	5.3	5.3
Equatorial Guinea	GNQ	4.2	15.8	0.03	45.2	45.2
Ethiopia	ETH	3.7	1.4	0.03	9.6	9.6
Finland	FIN	9.7	3.1	0.1	0.2	0.2
French Guiana	GUF	65.4	79.1	0.5	5.7	5.7
Gabon	GAB	108.8	41.2	0.8	22.9	22.9
Georgia	GEO	9.0	18.3	0.1	0.7	0.7
Guatemala	GTM	5.7	5.2	0.04	13.3	13.3
Guyana	GUY	144.1	69.6	1.1	11.3	11.3
Honduras	HND	6.7	6.0	0.1	28.6	28.6
India	IND	33.7	5.6	0.3	1.6	1.6
Indonesia	IDN	359.2	20.1	2.8	10.8	10.8
Japan	JPN	1.2	0.4	0.01	0.01	0.01
Kazakhstan	KAZ	4.4	16.6	0.03	2.3	2.3
Laos	LAO	8.5	3.8	0.1	47.9	47.9
Liberia	LBR	4.7	5.0	0.04	32.2	32.2
Madagascar	MDG	17.2	7.2	0.1	19.0	18.5
Malaysia	MYS	21.1	6.5	0.2	25.1	25.1
Mexico	MEX	15.0	1.8	0.1	2.8	2.6
Mongolia	MNG	11.7	12.6	0.1	12.5	0.4
Myanmar	MMR	52.9	10.1	0.4	30.9	30.9
Nepal	NPL	0.6	0.6	0.004	0	0
New Zealand	NZL	43.1	25.4	0.3	1.3	1.2
Nicaragua	NIC	10.3	8.0	0.1	38.1	38.1
Nigeria	NGA	3.0	1.3	0.02	5.3	5.3
Norway	NOR	1.8	1.4	0.01	1.0	1.0
Panama	PAN	14.5	19.6	0.1	19.8	19.8
Papua New Guinea	PNG	159.8	35.1	1.2	13.3	13.3
Paraguay	PRY	44.5	11.1	0.3	79.3	79.3
Peru	PER	567.2	68.5	4.4	6.1	6.1
Philippines	PHL	4.0	1.6	0.03	9.5	9.5
Republic of the Congo	COG	138.7	40.7	1.1	17.7	17.7
Romania	ROU	1.0	0.6	0.01	100.0	100.0
Russia	RUS	2744.3	28.3	21.4	6.5	4.3
Samoa	WSM	0.7	23.8	0.01	0.6	0.6
Solomon Islands	SLB	7.8	32.3	0.1	52.9	52.9
Suriname	SUR	107.4	73.8	0.8	5.7	5.7
Sweden	SWE	11.6	3.0	0.1	0.8	0.8
Tanzania	TZA	4.1	0.8	0.03	2.3	2.3
Thailand	THA	19.4	7.0	0.2	7.8	7.8
Uganda	UGA	1.0	0.7	0.01	0.9	0.9
United States	USA	539.3	14.2	4.2	7.9	0.2
Vanuatu	VUT	0.7	7.5	0.01	1.1	1.1
Venezuela	VEN	312.8	35.7	2.4	1.5	1.5
Vietnam	VNM	4.1	1.7	0.03	25.5	25.5

Globally, 30% of the world forest area (land with tree canopy cover of 20% or greater) was within IFLs in the year 2000. Most of the IFL area (82.3%) is covered with forest. The rest is covered with intact treeless ecosystems (montane grasslands, treeless wetlands, and burned areas as a consequence of wildfires) and a small fraction of nonvegetated areas (water, rocks, and ice).

**Fig. 2:**
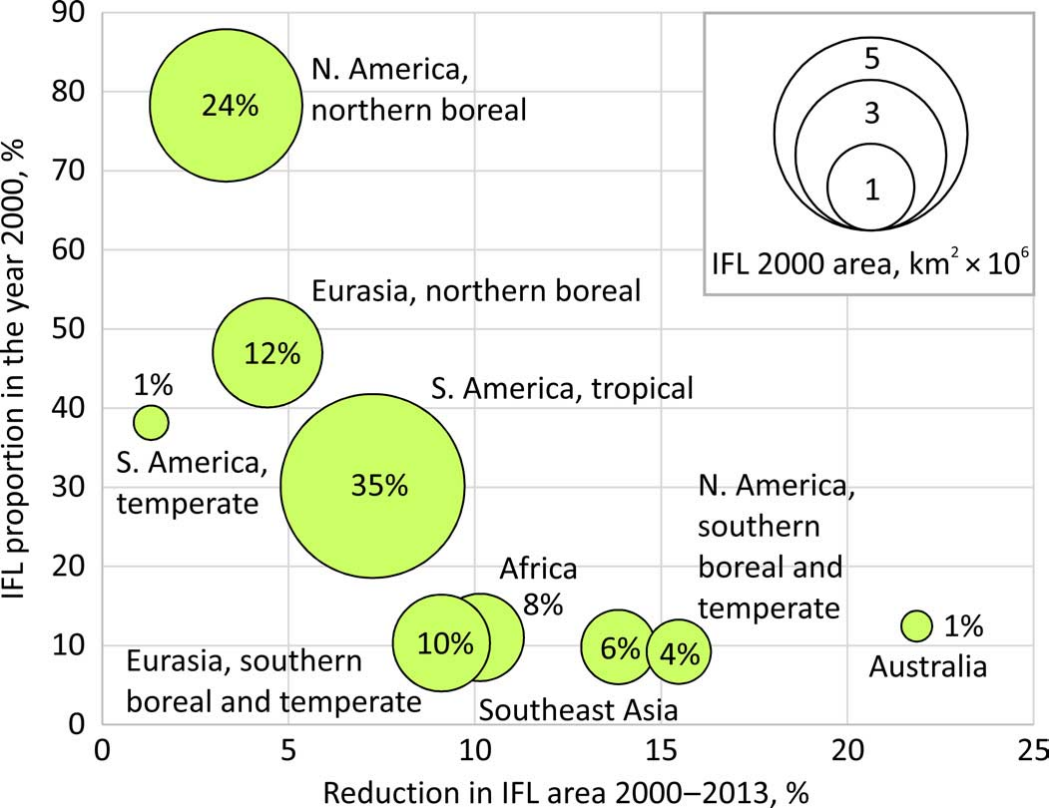
Distribution of IFL area in the year 2000 and reduction of IFL area 2000–2013 by geographic region. The *y* axis shows the initial IFL proportion of the forest zone in the year 2000. The *x* axis shows the reduction in IFL area from 2000 to 2013 as the proportion of IFL 2000 area. The area of each bubble indicates the IFL area in km^2^ × 10^6^. Values within each bubble represent the regional IFL area in the year 2000 as a percent of the global total.

From 2000 to 2013, the global IFL area decreased by 7.2%, a reduction of 919,000 km^2^ (Table 1). Tropical regions are responsible for 60% of the total reduction of IFL area. In particular, tropical South America lost 322,000 km^2^ of IFL area, whereas Africa lost 101,000 km^2^. Temperate and southern boreal regions contributed 21% to the global IFL area loss. Northern Eurasia alone lost 112,000 km^2^ of its IFL area. The remaining 19% of IFL area reduction occurred within the northern boreal forests of Eurasia and North America. Compared to the year 2000 IFL extent, the proportion of the IFL area reduction was lowest in the northern boreal regions and in the temperate forests of South America and highest in Australia, Southeast Asia, Africa, and the temperate regions of North America and Eurasia (Fig. 2).

**Fig. 3:**
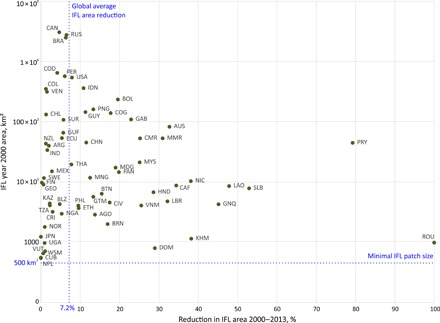
Distribution of IFLs by country in the year 2000 and reduction of IFL area 2000–2013. The *y* axis shows the IFL area in the year 2000. The *x* axis shows the reduction in IFL area from 2000 to 2013 as the proportion of IFL 2000 area. Country codes are given in Table 2.

Three countries comprise 52% of the total reduction of IFL area: Russia (179,000 km^2^ of IFL area lost), Brazil (157,000 km^2^), and Canada (142,000 km^2^). Proportional to the year 2000 IFL area, the highest percentages of IFL area reduction were found in Romania, which lost all IFLs, and Paraguay, where 79% of IFL area was lost; Laos, Equatorial Guinea, Cambodia, and Nicaragua each lost more than 35% of their IFL area (Fig. 3 and Table 2). Assuming that the loss of IFLs continues at the average rate between 2000 and 2013, Paraguay, Laos, Cambodia, and Equatorial Guinea will lose their entire IFL area during the next 20 years. Another 15 countries will lose all IFLs within a 60-year period, including such IFL-rich nations as the Republic of the Congo, Gabon, Cameroon, Bolivia, and Myanmar.

**Fig. 4:**
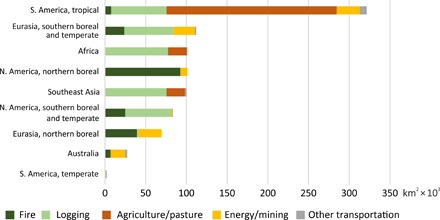
Regional reduction of IFL area (km^2^ × 10^3^) and causes of change.

We used stratified sampling to identify the primary causes of the IFL area reduction. At the global level, the leading fragmentation and alteration agents were timber harvesting (37.0% of global IFL area reduction), agricultural expansion (27.7%), and wildfire spread from infrastructure and logging sites (21.2%). Other causes included fragmentation by roads for mining and oil/gas extraction, pipelines, and power lines (12.1%) and expansion of the transportation road network (2.0%). At the regional level, we observed a diversity of leading IFL area reduction causes (Fig. 4 and Table 3), whereas for each particular region, a single cause accounted for more than 50% of the regional IFL area reduction.

**Table 3:** Sample-based assessment of the causes of IFL area reduction.

	**Total IFL** **area reduction** **(km^2^ × 10^3^)**	**Number** **of samples** **(1 km^2^ each)**	**The IFL area reduction by proximate cause, km^2^ × 10^3^ (standard error, km^2^ × 10^3^)**				
			**Wildfire**	**Timber** **harvesting**	**Agriculture and** **pasture expansion**	**Mining, oil** **and gas,** **hydropower**	**Other transportation,** **tourism**
Africa	101.3	100	0	77.5 (0.4)	22.8 (0.4)	0	1.0 (0.1)
Australia	27.4	50	6.6 (0.2)	0	0.5 (0.1)	17.6 (0.2)	2.7 (0.1)
South America, temperate	2.1	50	0.5 (0.01)	0.9 (0.01)	0	0	0.7 (0.01)
South America, tropical	321.5	300	7.5 (0.3)	68.1 (0.8)	209.0 (0.9)	28.4 (0.5)	8.6 (0.3)
North America, temperate and southern boreal	83.3	84	24.8 (0.4)	56.6 (0.4)	0	1.0 (0.1)	1.0 (0.1)
North America, northern boreal	101.2	116	92.5 (0.3)	0	0	8.7 (0.3)	0
Northern Eurasia, temperate and southern boreal	112.1	113	23.8 (0.4)	60.0 (0.5)	0	26.3 (0.4)	2.0 (0.1)
Northern Eurasia, northern boreal	69.5	87	39.2 (0.4)	1.6 (0.1)	0	28.8 (0.4)	0
Southeast Asia	100.2	100	0	75.6 (0.4)	22.6 (0.4)	0	2.0 (0.1)

**Fig. 5:**
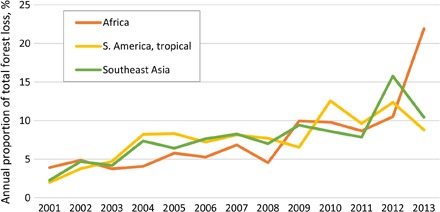
Annual proportion of the total forest loss within tropical forests that lost IFL status between 2000 and 2013.

Using sample-based analysis and the annual forest loss data set (*18*), we found that 14% of the total IFL area reduction was due to direct alteration caused by logging, clearing, and fires. The remaining 86% was due to fragmentation by such disturbances and construction of infrastructure. The annual forest loss within IFLs may be used as a proxy to understand the temporal dynamics of IFL area reduction. In tropical regions, the annual forest loss within IFLs increased during the past 13 years (Fig. 5). The average annual forest loss within IFL reduction area for the 2011–2013 period was triple the average for the 2001–2003 period for each of the three tropical regions, with the highest increase observed in central Africa.

Of the total IFL area in the year 2000, 12.4% fell within protected areas (PAs), with a management regime consistent with the International Union for Conservation of Nature (IUCN) categories I to III (*19*). Australia and temperate South America have the largest proportion of IFLs under legal protection (47.4 and 43.7%, respectively), whereas temperate and southern boreal northern Eurasia (7.7%) and northern boreal regions (7.7% in North America and 5.2% in Eurasia) have the lowest. Forty of the 65 countries, in which IFLs were present in the year 2000, had at least 10% of the IFL area under legal protection. Uganda, the Dominican Republic, Thailand, and Cuba had protected more than 90% of their IFL area. Some countries do not include any IFLs within category I to III PAs, including many Southeast Asian countries (Lao People’s Democratic Republic, Vietnam, Cambodia, and Philippines), Papua New Guinea, Ethiopia, Angola, and Nicaragua.

Using matching sampling analysis, we found that the reduction of IFL area for reasons other than fire was 3.4 times higher outside PAs (6.2%) than within PAs (1.8%). We found a large difference in most regions between protected and unprotected areas in terms of IFL area reduction (Table 4). In Africa, North America, and Eurasia, the reduction of IFL area was more than 4 times higher outside PAs than inside PAs, whereas it was 2.6 times higher in Southeast Asia and almost double in tropical South America.

**Table 4:** IFL area reduction inside and outside IUCN category I to III PAs. Area-based estimate represents area calculated from the map. Sample-based estimate is based on matching sampling analysis performed only within portions of IFLs vulnerable for degradation. This analysis only considers the reduction of IFL area 2000–2013 that was not attributed to fire.

**Region**	**Area-based estimate**			**Sample-based estimate and standard error (SE)**	
	**IFL 2000 within** **IUCN category** **I–III PAs (%)**	**IFL area reduction** **within PAs (%)**	**IFL area reduction** **outside PAs (%)**	**IFL area reduction** **within PAs, % (SE, %)**	**IFL area reduction** **outside PAs, % (SE, %)**
Africa	10.8	1.6	11.2	5.5 (0.72)	25 (1.37)
Australia and New Zealand	47.4	9.6	20.5	54.6 (1.57)	44.1 (1.57)
Temperate South America	43.7	0.4	1.3	1.6 (0.40)	1.1 (0.33)
Tropical South America	15.1	2.0	8.0	8.0 (0.86)	14.6 (1.12)
Temperate North America	34.0	1.1	16.4	5.2 (0.70)	24.6 (1.36)
Temperate Northern Eurasia	7.7	1.4	7.9	3.2 (0.56)	17.5 (1.20)
Southeast Asia	12.7	4.6	15.2	6.8 (0.80)	17.9 (1.21)

To study the effect of legal protection and voluntary forest management certification on IFL area reduction by logging, we analyzed PAs and timber concessions in the three central African countries, where up-to-date spatial information on forest management exists: Cameroon, Gabon, and the Republic of the Congo. Some of the concessions were certified to the Forest Stewardship Council (FSC) standard. Certified concessions had the same or higher proportion of IFL area reduction than noncertified concessions, whereas the IFL area loss was at least four times lower in PAs than in timber concessions (Table 5).

**Table 5:** IFL extent and area reduction within logging concessions in three central African countries. The spatial database of logging concessions in Cameroon (2013), Republic of the Congo (2013), and Gabon (2012) was obtained from the World Resources Institute (www.wri.org/our-work/project/congo-basin-forest-atlases).

**Country**	**IFL proportion** **of total concession** **area in 2000 (%)**	**IFL proportion** **of FSC-certified** **concession area** **in 2000 (%)**	**IFL area** **reduction** **2000–2013** **within the** **country (%)**	**IFL area** **reduction** **2000–2013** **within all** **concessions (%)**	**IFL area** **reduction** **2000–2013** **within FSC-certified** **concessions (%)**	**IFL area** **reduction** **2000–2013** **within PAs** **(IUCN category I–III) (%)**
Cameroon	40.5	38.4	25.2	41.1	84.5	0.3
Republic of the Congo	42.4	61.6	17.7	37.1	41.9	4.8
Gabon	48.4	29.7	22.9	37.9	37.0	9.0

## DISCUSSION

### Causes of IFL area reduction

Industrial timber extraction, resulting in forest landscape alteration and fragmentation, was the primary global cause of IFL area reduction. In Africa and Southeast Asia, selective logging was the dominant IFL loss cause (77 and 75% of the total loss of IFL area, respectively), whereas clear-cutting was the main IFL loss cause in the temperate and southern boreal regions of North America and Eurasia (68 and 54%, respectively). The relative proportion of forest loss and fragmentation within IFL reduction area depends on the logging method and the intensity of timber extraction. Clear-cuts caused a higher proportion of forest alteration (15% of the total IFL area reduction) compared to selective logging (1.2%), with the remaining IFL reduction attributed to fragmentation by logging sites and roads. Southeast Asia had a higher proportion of clearing within selectively logged areas than tropical Africa and South America (1.4 versus 0.3% for each of the latter).

**Fig. 6:**
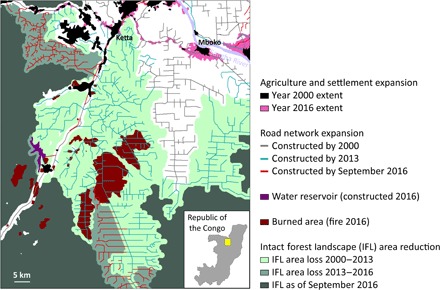
Stages and causes of the IFL area reduction and landscape transformation in the Republic of the Congo (map center at 16°0′E 1°12′N). The infrastructure and IFL extent within the area are shown as of September 2016. The map shows expansion of settlements and regional transportation and logging roads from the year 2000 until 2016. Logging road expansion caused the reduction of IFL area. IFL extent was mapped for the years 2000, 2013, and 2016. New settlements and agricultural areas appeared along existing and established roads. Logging expansion triggered forest fires that initiated from the roads and forest clearings. In September 2016, a water reservoir was constructed within the remaining IFL area, which caused continuous fragmentation and transformation of the surrounding landscape.

Expansion of logging into intact forest areas has many direct effects on ecosystem functions, including reduction of carbon storage (*20*), decrease of habitat suitability (*6*, *21*), and increase of vulnerability to human-induced wildfires (*22*, *23*). Fragmentation of forest landscapes by logging and logging roads causes direct habitat loss (*24*) and increases the incidence of poaching (*25*), resulting in species loss. Even within areas designated for sustainable forest management, like some tropical timber concessions, the construction of new logging roads initiates a cascade of land use changes and subsequent reduction in landscape conservation value. The example from the Republic of the Congo (Fig. 6) shows how expansion of logging infrastructure and a new hydropower project have markedly reduced IFL area. Agricultural expansion, forest fires, and the potential increase of unregulated hunting (*26*) are coincident with the expansion of the logging road network.

Agricultural expansion was the second most important cause of IFL area reduction. In tropical South America, expansion of agriculture overall and of pastures in particular contributed 65 and 53% of the overall IFL area loss, respectively. Expansion of industrial crops (for example, soybean) was not detected as a cause of IFL area reduction using our sample-based analysis. IFLs were not directly affected by industrial crop expansion in South America because it mainly occurred in areas previously converted to pastures (*27*). In tropical Africa and Southeast Asia, slash-and-burn smallholder agricultural expansion contributed 23 and 15%, respectively, to the total IFL area reduction.

**Fig. 7:**
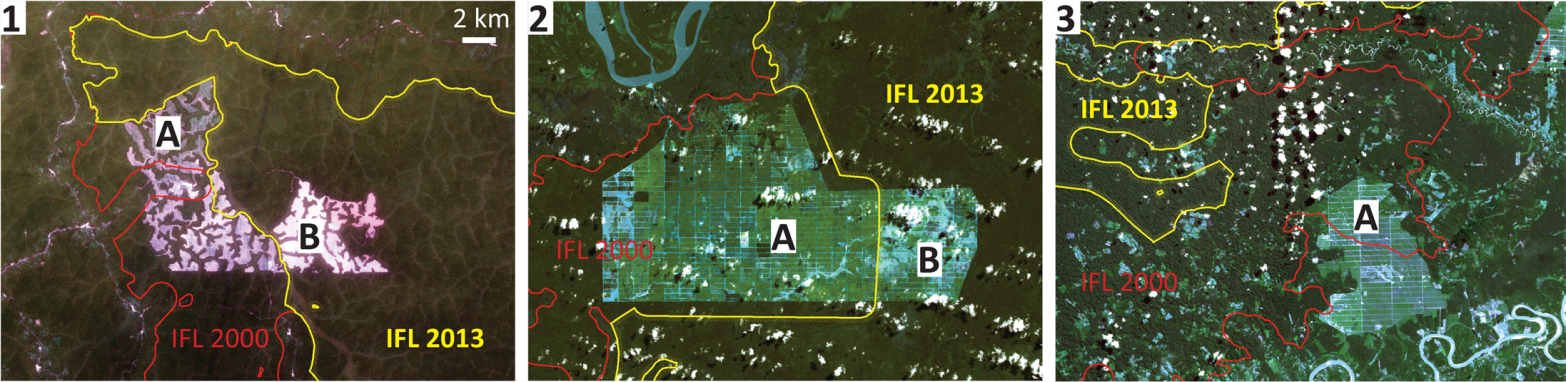
Examples of the ongoing expansion of oil palm plantations within IFLs in tropical regions. Each example shows IFL degradation depicted in year 2015 or 2016 cloud-free Landsat-8 satellite images. All maps have the same scale. The IFL boundary in 2013 is marked by a yellow line, and the IFL boundary for the year 2000 is marked by a red line. Oil palm plantations established before the year 2013 are denoted as “A,” and plantations established after 2013 are denoted as “B”. (1) Gabon; image subset centered at 11°47′E 2°7′N; image date, 12 January 2015. (2) Indonesia; subset centered at 139°45′E 7°21′S; image date, 10 May 2016. (3) Peru; subset centered at 75°7′W 8°15′S; image date, 24 June 2016.

Establishment of oil palm plantations contributed 0.2% of the total IFL area reduction. We found new oil palm plantations affecting IFLs in all tropical regions (Fig. 7). Plantations usually follow selective logging expansion and represent an example of how industrial logging operations can set off a cascade of interventions that eventually result in the final conversion of natural forests to industrial monoculture plantations (*28*).

Forest fires associated with infrastructure and therefore assumed to be human-induced accounted for 21% of the total IFL area reduction. Fire-related degradation was found within all regions except Southeast Asia. The absence of fires as an IFL degradation cause in Indonesia is explained by the fact that the remaining IFLs are located within remote mountain areas, whereas fires are much more prevalent in fragmented and degraded lowland forests. Fire was the main cause of IFL area reduction in northern boreal regions (91% in North America and 56% in northern Eurasia) and accounted for more than 20% of IFL reduction in temperate North America, temperate Eurasia, and Australia. Excluding fires as a cause of IFL degradation would change the global IFL area reduction from 7.2 to 5.7% (Table 1) but would not lead to notable changes in the ranking of regions by the proportion of IFL area lost.

Energy production (oil and gas extraction and hydropower) and mining operations are globally important causes of IFL area reduction due to the fragmenting effect of their transportation infrastructure. Oil and gas extraction was the leading fragmentation cause in northern Eurasia (specifically in the Russian Federation), accounting for 41% of IFL reduction in the northern boreal and 23% in the southern boreal and temperate forests. Russia is the largest producer of crude oil and the second largest producer of natural gas in the world. Recent expansion of oil and gas exploration and extraction in East Siberia caused fragmentation of the forest wildlands through establishment of new pipelines and extraction infrastructure, usually accompanied by logging and human-ignited fires. Mining and mineral exploration (mostly for gold) played a significant role in Australia (64% of the total IFL reduction) and tropical South America (9%).

Fragmentation generally dominates over forest clearing as a factor of IFL area reduction. Their relative contribution depends on the type of disturbance. The highest percent of forest clearing was observed for oil palm plantation establishment (43% of the total IFL area reduction) and forest fires (41%), followed by clear-cut logging (15%), pasture (15%), and other agricultural expansion (8%). However, the core areas of IFL also experience natural forest loss events. Intact landscapes are not static in terms of land cover change. Large-scale wildfires, pest attacks, and wind damage occur naturally in many temperate and boreal forests, where they are followed by natural regeneration. According to the global forest cover loss product (*18*), the total area of forest loss within IFL from 2001 to 2013 was 314,000 km^2^, or 2.5% of the IFL area. This includes both anthropogenic and natural disturbances. The IFL 2000–2013 change data set shows that 55% of the total forest loss area fell within stable IFL areas and was therefore assumed to represent natural ecosystem dynamics. However, for the tropical regions, the proportion of natural disturbance within IFLs was small (8.6% of the total forest loss area within year 2000 IFLs).

### Legal protection of IFLs

In all regions, the proportion of the reduction of IFL area was lower inside of PAs than outside of PAs (Table 4), suggesting that legal protection was effective in preventing IFL loss. However, this conclusion may be invalid due to the nonrandom distribution of PAs within IFL areas (*29*). To control for the varying vulnerability of IFLs to human alteration and fragmentation, we implemented a sample matching method to account for the nonrandom distribution of PAs. The results confirmed that legal protection has been effective at lessening the reduction of IFL area in all regions except Australia (where roads have been constructed near PA boundaries) and temperate South America (where new tourist infrastructure has been developed in a national park). However, when analyzing the causes of IFL area reduction, we noticed that legal protection was not always an effective way to limit agricultural expansion. Of the 10 PAs in Africa, classified as IUCN categories I and II that experienced more than 1% IFL area loss, 7 were subjected to smallholder agricultural expansion. Two of these PAs are in Andasibe-Mantadia National Park (in which all IFLs disappeared) and Tsaratanana Strict Nature Reserve (in which 28% of the IFL area was lost). In both cases, slash-and-burn agriculture expanded within park boundaries. The same process was observed in Virunga National Park (Democratic Republic of the Congo), which lost 3.3% of its IFL area due to agricultural expansion.

Another cause of IFL area reduction within PAs is the development of new infrastructure. In some cases, new transportation infrastructure causes fragmentation, as in Domogled-Valea Cernei National Park (Romania). In other cases, the development of infrastructure for tourism and recreation caused IFL area reduction, for example, the expansion of the road network in Puyehue National Park (Chile) and the construction of a ski resort within Sochinsky National Park (Russia). Although some of these infrastructure projects were developed to increase PA income and stimulate public awareness of the importance of nature conservation, they nevertheless had the effect of reducing the extent of remaining forest wildlands through fragmentation.

Many IFLs contain high-value timber resources, and logging and associated fragmentation by roads are the leading causes of IFL area reduction worldwide. Standards for responsible forest management, including those of the FSC, seek to balance forest-based economic development with conservation. FSC regards IFLs as a type of high conservation value forest, and the FSC standard states that their degradation should be avoided. In 2014, the General Assembly of FSC adopted a motion (Motion 65) that calls upon FSC to do the following: “within IFL cores ensure that Certificate Holders implement protection measures (for example, set-asides, legal protected areas, conservation reserves, deferrals, community reserves, indigenous protected areas etc.) ensuring management for intactness” (*30*). If Motion 65 is implemented, we should, at least, in the future, expect IFL fragmentation to proceed more slowly within FSC-certified concessions than in noncertified concessions. Our results from the period 2000–2013 suggest that the pace of IFL fragmentation due to selective logging in central Africa is faster within FSC-certified concessions than outside them, due to selective logging and fragmentation by logging road construction (Table 5). By definition, selective logging and establishment of associated infrastructure in an IFL reduce its area. Although we do not know the degree to which IFL fragmentation is actively avoided by logging operations, it is evident that selective logging within FSC-certified concessions is a significant driver of IFL area reduction in central Africa. For other regions, sufficiently detailed spatial information on logging concessions and certification is largely unavailable, precluding similar analysis.

### Regional approaches to IFL monitoring

National projects focused on characterizing “primary forests,” “high conservation value forests,” or “wilderness areas” are complementary to the global IFL mapping initiative. Such maps often provide information on smaller fragments of high conservation value forests located outside of the largest wilderness areas. The work of Global Forest Watch Canada (GFWC) represents an example of regional IFL mapping that uses different criteria from our global method. GFWC criteria allow for inclusion of all burned areas within IFLs, regardless of the cause of fire, and require a smaller minimum area for a patch to qualify as an IFL (*31*, *32*). The GFWC IFL map has been updated for the year 2013 (*33*), allowing for a comparison of regional and global IFL maps. The GFWC map for 2013 showed that Canada has a total IFL area that is 1.4 times larger than the one shown in our global map. However, 98.6% of the intact area from our global map is included in the GFWC map, illustrating agreement on the location and extent of core wilderness areas.

The standard method presented in this paper is capable of providing a globally consistent characterization of the extent of IFLs and its change over time. However, for regional mapping initiatives, regional relevance may be a higher priority than global consistency. Regional assessments may wish to deviate from the standard global method by using criteria that are adapted to the regional context, as GFWC does. It is important to be clear on the differences in criteria as they may explain a major part of the seeming discrepancy between a regional and a global map.

An important difference between the global IFL assessment presented here and the regional IFL assessment produced by GFWC is the treatment of fire-related disturbances. It is typically not possible to determine whether a fire had a natural origin or was caused by people. In the global assessment, burned areas in the vicinity of transportation infrastructure, agricultural areas, and logging sites were assumed to be caused by humans and thus were treated as an IFL reduction factor. Although lightning strikes can ignite forest fires, several studies have found that most fires in the vicinity of infrastructure and logging sites are of human origin, in boreal (*22*, *34*) as well as in temperate (*35*, *36*) and tropical forests (*37*). However, large fires may be of natural origin even if colocated with infrastructure (*38*, *39*). Our approach has been to construct a set of mapping rules that can be applied consistently at the global scale. For burned areas, our rule assumes that fires in the vicinity of areas with human access are likely to have a human cause. Regional conservation specialists (*40*) have challenged the utility of applying globally consistent criteria at regional scales, specifically in interpreting the causes of fires in boreal Canada. In response to these concerns, our global analysis differentiates IFL reduction due to fire from other causes.

The IFL concept is defined to map the large unfragmented tracts of primary forests. A different set of criteria, using a smaller threshold for minimum patch size, would be needed to map small fragments of primary forest. Our earlier work in central Africa and insular Southeast Asia showed that substantial areas of primary forests exist outside of IFLs. We found that 38.6% of the primary forest area in the Democratic Republic of the Congo (*41*) is located outside of IFLs, whereas on the island of Sumatra, Indonesia, the proportion is 73.2% (*28*). The method presented here can be used to identify conservation priority areas at the regional and national levels if the criteria for minimum eligible patch size and alteration are adjusted for this purpose.

### Accuracy of the global IFL map

To assess the accuracy of the IFL 2000–2013 change map, we used the same 1000 random samples that were used to assess the causes of IFL area reduction. The samples were interpreted separately from the generation of the map. The sampling design made it possible to estimate commission error (that is, change that had been falsely attributed to human causes) but not omission error (human-caused change that had been overlooked, that is, that was not reflected in the change map). Visual interpretation of Landsat imagery and of high-resolution imagery available through Google Earth confirmed that 92% of the sampled area of IFL area reduction had been correctly classified. It was not possible to confirm whether the alterations for the remaining sampled area (8%) were human-caused based on Landsat or high spatial resolution satellite imagery.

A partial field validation of the IFL 2000 map by Greenpeace Russia and GFWC (*42*, *43*) confirmed that intact areas within the boreal and temperate forests of European Russia and Canada had been correctly classified. An alternative approach to validation focused on forest structure to differentiate intact forests from forests within degraded or altered landscapes. Studies by Margono *et al.* (*28*) and Zhuravleva *et al.* (*41*) used data from the Geoscience Laser Altimeter System to examine the tree canopy structure inside and outside of IFLs in Sumatra (Indonesia) and the Democratic Republic of the Congo. Their results revealed a statistically significant difference in average forest height between intact forests and other forests (fragmented and altered).

### IFL role in climate change mitigation

The primary forests that remain within IFLs represent the most significant carbon pool within the tropical biome (*44*). Using a benchmark tropical forest carbon map produced for the early 2000s (*45*), we estimate that the total biomass carbon pool in the tropical forest zone was 243 Gt C around year 2000, of which IFLs stored 97 Gt C (40%). The average carbon density was greater in IFLs than in the rest of the tropical forest zone: 3.7 times higher in Africa, 3.4 times higher in South America, and 1.7 times higher in Southeast Asia.

IFLs in the boreal and temperate regions differ from those in the tropics by having lower biomass per unit area and lower productivity than managed forests. In the year 2000, the average growing stock in North America and Eurasia was 1.4 times higher in forests outside IFLs (145.5 m^3^/ha) than within IFLs (103.1 m^3^/ha) (*46*). This has historical reasons. In the past, temperate and southern boreal forests have been cleared, converted into managed forests, or fragmented by infrastructure, leaving mostly low productivity forests (specifically, peatlands and mountains) as IFLs (*42*). Nevertheless, the vast areas of boreal IFLs represent a large and relatively stable above- and belowground carbon storage that plays an important role in the global climate system. Although the recent increase in boreal wildfire frequency and intensity (*39*) threatens long-term aboveground carbon storage in northern forests, it has been shown that IFLs have a lower fire frequency compared to fragmented and developed areas (*22*). Permafrost protection is another important IFL function. Road and pipeline constructions have multiple direct and indirect effects on permafrost, increasing its vulnerability to thawing (*47*). Almost 52% (2.6 million km^2^) of the total continuous and discontinuous permafrost area within forest zone in North America and Eurasia is located within the remaining IFLs (*48*).

## CONCLUSIONS

Intactness is a good indicator of the comprehensive conservation value of a forest landscape (*7*, *8*). It is related to specific ecosystem values, such as ecosystem integrity and resilience to natural disturbances and to ongoing climate change. It is also related to other forest ecosystem functions, such as biodiversity (*49*). It can be reduced very rapidly, in a matter of months and years, by increased fragmentation and access, even without changes in tree canopy cover. On the other hand, intactness is hard to gain, at least within a short time span. That is why intact landscapes should be treated as having high (or even the highest) conservation value. The conservation value of an intact area is dependent on its size because many umbrella mammal and bird species require large natural habitats to survive (*12*, *50*). That is why the size of the intact area should always be taken into consideration when assessing wildland conservation value. The Congress of the IUCN held in Hawaii in 2016 adopted a motion (Motion 048) that “encourages states, the private sector and international financial institutions to: a. avoid loss and degradation of primary forests, including intact forest landscapes; b. promote conservation of primary forests, including intact forest landscapes” (*51*). National approaches to protecting IFLs include expansion of the PA network and the establishment of a system for wilderness area management similar to that of the United States (*52*). Large forested wildlands often straddle international boundaries, highlighting the need for effective international conservation strategies (*10*). IFLs provide a framework for maintaining large, contiguous, and often transnational blocks of forest wildlands. The high carbon stocks found within IFLs illustrate their potential benefit to climate change mitigation strategies. This study has demonstrated that legal protection is an effective policy for reducing the degradation of IFLs. We suggest that IFLs should be considered when existing PA networks are revised and expanded. We also suggest that monitoring of forest intactness should be treated as an important aspect of national and global forest assessments.

## MATERIALS AND METHODS

The extent of the forest zone was mapped using the global year 2000 tree canopy cover data set (*18*) with a 20% tree canopy cover threshold. Inland water bodies and naturally treeless ecosystems were included in the forest zone. Fragments of land in the forest zone with a contiguous area smaller than 500 km^2^ were excluded from consideration. Geographic regions within the forest zone (Fig. 1) were delineated using natural boundaries between forested areas. The boundary between northern boreal and southern boreal/temperate regions in North America and northern Eurasia was based on Landsat data analysis and represents the de facto dividing line between lands that have, and have not, been subjected to industrial logging as of the year 2013.

An IFL is defined as a seamless mosaic of forests and associated natural treeless ecosystems that exhibit no remotely detected signs of human activity or habitat fragmentation and is large enough to maintain all native biodiversity, including viable populations of wide-ranging species (*15*). An IFL includes both forest and naturally treeless ecosystems. Two main criteria were used to distinguish an IFL patch from the surrounding landscape: (i) ecosystem alteration and (ii) landscape fragmentation by infrastructure and disturbance. Areas that have been altered or managed (through agriculture, logging, and mining) were excluded, along with a buffer zone of 1 km (*53*) on either side of infrastructure elements (roads, pipelines, power lines, and navigable rivers). Past disturbances that occurred more than 30 to 70 years ago, scattered small-scale shifting cultivation, nonindustrial timber harvesting by indigenous forest dwellers, and low-intensity disturbance not directly observable in remotely sensed data (hunting and forest grazing) were not considered IFL alteration or fragmentation factors. An IFL patch must have (i) a minimum size of 500 km^2^, (ii) a minimum width of 10 km, and (iii) a minimum corridor/appendage width of 2 km. Any patch that falls below these thresholds, for example, due to fragmentation, logging, or fire, was rejected in its entirety.

Source data for IFL mapping and monitoring were taken from the global archive of medium spatial resolution Landsat satellite imagery. We used a collection of single-date Landsat images (*15*) to map IFLs for the year 2000. Landsat images circa year 1990 were used to map forest disturbances in the tropics that may be invisible in the images from the year 2000 without previous knowledge. For the year 2013 IFL update, we used seamless, cloud-free Landsat data composites and a Landsat-based annual forest loss product (*18*). IFL mapping for 2000 and 2013 was performed using visual interpretation of Landsat imagery. A number of ancillary data sources were used to assist interpretation, including national transportation maps, existing forest cover change products, and high-resolution remotely sensed data from Google Earth. We used an “inverse logic” approach to delineate IFLs. Initially considering the entire forest zone as a candidate for IFL status, we systematically identified and eliminated altered and fragmented areas until all available evidence had been exhausted. We then attributed the remaining unfragmented portion of the forest zone that fit our size criteria as an IFL. When estimating the reduction in IFL area between 2000 and 2013, we rejected all patches that fell below the threshold of intactness during this period, even when only by a small margin. Thus, a patch of 800 km^2^ that was bisected by a road into two patches of 400 km^2^ each would register as a reduction in area of 800 km^2^.

To identify the causes of IFL area reduction, we used a sampling approach based on a stratified random design. We allocated a total of 1000 IFL area reduction samples, each 1 km^2^ in size, among the IFL regions (Fig. 1) in proportion to each region’s IFL area reduction in absolute terms (Table 1). For each sample, we examined the cause of both IFL reduction and forest loss using all available remotely sensed data (annual Landsat data composites, data from Google Earth).

To estimate the effectiveness of legal protection as a means for reducing the loss of IFL area, we used a matching sampling approach to account for the nonrandom distribution of PAs. To account for factors that influence the probability of IFL area reduction, we used the following metrics: (i) elevation (*54*), (ii) slope, (iii) distance to IFL boundary, (iv) tree canopy cover for the year 2000, and (v) the human footprint index (*3*). In each country and ecozone, we assessed the distribution of these metrics on areas that had lost IFL status between 2000 and 2013, allowing sampling plots to be selected only where the value of each variable was within ±1 SD of the mean, that is, in areas with a high probability of change. In each geographic region, we randomly allocated a set of 1000 samples of 1 ha each within the protected portion of the IFLs (IUCN categories I to III) (*19*). We then selected the closest matching sample from the unprotected portion of the IFLs in the same country (Global Administrative Areas Database, http://gadm.org) and in the same ecozone (*55*) using the Euclidean distance in metric space. As a result, two matching populations of samples (protected and unprotected) were obtained for each region. The differences in the sample-based IFL area change rate from these two populations were used as an unbiased measurement of IFL reduction within and outside PAs.

To analyze the effect of FSC certification on IFL area reduction in selected central African countries, we used the logging concession database collected by the World Resources Institute (www.wri.org/our-work/project/congo-basin-forest-atlases). The logging concession spatial database for three countries was used: Cameroon (database for year 2013), Republic of the Congo (2013), and Gabon (2012).
